# Musculoskeletal Disorders, Healthy Buildings, and the Hotel Industry: A Narrative Review and Critical Reflection

**DOI:** 10.7759/cureus.102991

**Published:** 2026-02-04

**Authors:** Ezequiel D Gherscovici, John M Mayer

**Affiliations:** 1 Research and Development, Healthy Buildings LLC, Malibu, USA

**Keywords:** delivery of health care, healthy buildings, hotels, musculoskeletal disorders, occupational health

## Abstract

Musculoskeletal disorders (MSDs) continue to be problematic globally. Numerous guideline-based management approaches have been advocated for MSDs, but long-term recovery is elusive. Thus, innovations should be explored to help mitigate the adverse consequences of these disorders. The objective of this paper was to provide a narrative literature review and critical reflection of MSDs and healthy buildings in the hotel industry. Searches of the peer-reviewed medical literature (via PubMed) and hospitality literature (via SCImago) were conducted for MSDs, healthy buildings, and hotels. The available evidence from the searches was synthesized and critically examined, and research and implementation recommendations were proposed. Key findings include the available evidence displays a substantial risk of bias, and numerous gaps in knowledge and research exist at the aggregate interface of MSDs, healthy buildings, and hotels; hotel workers appear to be particularly vulnerable to MSDs, yet interventions are needed to address MSDs in this occupational setting; and interventions delivered in luxury resort hotels may be useful for guests with MSDs. This paper is the first known attempt to critically examine the new field intersecting MSDs, healthy buildings, and the hotel industry. In consideration of the limited available evidence, it offers recommendations for medical and hotel stakeholders to advance the field through scientifically sound research and implementation efforts. If positive findings are observed in future initiatives, a new healthcare value proposition is created, with the desired outcome of mitigating MSDs and improving the health, wellness, quality of life, and experiential takeaway of hotel employees and guests.

## Introduction and background

Rationale

Musculoskeletal Disorders

Musculoskeletal disorders (MSDs), such as back pain, neck pain, and extremity MSDs, are problematic and affect most types of people, occupational settings, and societies around the world [1-3]. Of the MSDs, back pain and neck pain are particularly burdensome and are among the most common, disabling, and costly health conditions [3]. In terms of years lived with disability, low back pain (#1) and neck pain (#6) outrank diabetes (#8), chronic obstructive pulmonary disease (#11), ischemic stroke (#17), and ischemic heart disease (#29) [3].

Numerous management approaches have been advocated for MSDs, which have displayed varying degrees of long-term success in improving recovery [4-9]. For MSDs, such as back pain and knee osteoarthritis, exercise and education are at the forefront of clinical practice guidelines [6,10], and these interventions are likely applicable across numerous settings. Considering the sustained burden of MSDs and the apparent lack of major breakthroughs in their management, exploring novel approaches, such as healthy buildings, could be advantageous [11].

Healthy Building Determinants

Healthy buildings are "a biopsychosocial framework that focuses on transforming the built environment to promote and enhance the health, wellness, performance, productivity, and quality of life of occupants" [11]. The healthy buildings movement is part of an ongoing initiative aimed at improving human health within the built environment. It has roots in ancient times [12], nineteenth-century and early twentieth-century efforts [13,14], sick building syndrome [15], and has recently evolved from green building concepts [16], utilizing technologies related to smart and intelligent buildings [17].

Healthy building determinants are "factors within the built environment that influence health status, wellness, performance, productivity, and quality of life of occupants" [11]. Several healthy building determinants have been discussed in the literature, including air quality and ventilation, dust and pests, lighting and views, moisture, noise, safety and security, thermal health, and water quality [18], including specifically in the context of MSDs [11,19]. Since people spend approximately 90% of their time indoors [20,21], targeting healthy building determinants could have a large impact on improving human health, wellness, and quality of life related to MSDs [11].

Three systematic reviews have examined the relationship between several healthy building determinants and MSDs [11,19,22]. As illustrated in Table 1, these reviews generally found weak evidence to support relationships between many healthy building determinants and MSDs. Namely, as these determinants worsen, the risk of MSDs increases. However, this research found minimal literature assessing interventions and no literature examining the relationships between healthy building determinants and MSDs within the hotel industry. Nonetheless, addressing healthy building determinants theoretically offers a solution to help combat MSDs.

**Table 1:** Summary of empirical evidence statements for the relationships of healthy building determinants with MSDs General MSDs: unspecified body region. Overall Work Environment: a combination of various healthy building determinants in the work environment. Empirical evidence statement descriptions: Weak: weak evidence to support a positive relationship between a healthy building determinant and MSDs. That is, as a healthy building determinant worsens, the risk of MSDs increases. Weak (against): weak evidence to support no relationship between a healthy building determinant and MSDs. Conflicting: conflicting evidence about the relationship between a healthy building determinant and MSDs. No Evidence: no evidence was uncovered to formulate an empirical evidence statement for the relationship. Table adapted from "Healthy Buildings and Musculoskeletal Disorders: Advances in Occupant Health and Wellbeing" [23]. Reprinted with permission from publisher: Healthy Buildings LLC, Malibu, California, USA. MSDs, musculoskeletal disorders

Healthy building determinant	Back pain	Neck pain	Upper extremity MSDs	Lower extremity MSDs	General MSDs
Air quality/ventilation	Weak	Weak	Weak	Conflicting	Weak
Dust and pests	Weak	No evidence	Weak	Conflicting	Conflicting
Lighting and views	Weak	Weak	Conflicting	Weak (against)	No evidence
Moisture	Weak	No evidence	Weak	Weak	No evidence
Noise	Weak	Weak (against)	Weak	Conflicting	Weak
Safety and security	No evidence	No evidence	Weak	No evidence	Weak
Thermal health	Uncomfortable: Weak; Cold: Weak; Warm: Weak (against)	Uncomfortable: Weak; Cold: Weak; Warm: Weak (against)	Uncomfortable: Weak; Cold: Weak; Warm: Conflicting	Uncomfortable: Weak; Cold: Weak; Warm: Weak	Uncomfortable: No evidence; Cold: Weak; Warm: Weak (against)
Water quality	Weak	No evidence	Weak	Weak (against)	Weak
Overall work environment	Weak	Weak	Weak	No evidence	Weak

Developing a detailed conceptual framework on how healthy building determinants may influence MSDs exceeds the available research evidence and is beyond the scope of this review. However, a plausible framework should involve identifying deficits for specific healthy building determinants and their relationships with MSDs, addressing these deficits via targeted interventions, and assessing the impact of these interventions on MSD outcomes [23]. For example, poor indoor air quality is related to an increased risk of low back pain [11,22]. Upgrading a building's air filtration and ventilation systems can improve indoor air quality and reduce exposure to air pollutants that affect health [24]. Furthermore, exercises to enhance breathing patterns can improve a person's ability to filter air and recover [25,26]. Theoretically, these improvements could result in reductions in pain and functional disability in patients with chronic low back pain [22,23].

Musculoskeletal Disorders in the Hotel Industry

The hospitality industry is among the top economic drivers globally [27]. The hotel industry, a sector within the hospitality industry, is large and growing [28,29], with the top 10 global hotel corporations owning nearly 50,000 properties [28]. Additionally, hotel utilization is projected to grow significantly, with demand for hotel rooms, occupancy rates, and average daily rates increasing worldwide [29]. Furthermore, hotels employ individuals who function in diverse occupational settings, with job demands ranging from sedentary to very heavy [30]. Considering its large influence, the hotel industry has the potential to play a significant role in the health, wellness, and quality of life of individuals it employs or who utilize its services as guests.

The hotel industry has recently embraced wellness concepts [31], yet the impact of this approach on MSDs is largely unknown. Two recent reviews provide some insight into the impact of MSDs on the hotel industry and useful information for future research. A 2024 systematic review and meta-analysis found that hotel housekeepers have high rates of MSDs [32], with the three most prevalent body regions being the low back (53.9%, 95%CI 43.3-64.6%), shoulder (41.4%, 95%CI 27.1-55.8%), and wrist/hand (40.1%, 95%CI 24.5-55.7%). A 2020 scoping review that assessed various health interventions for hotel workers [33] uncovered two small noncontrolled observational studies that showed benefits for musculoskeletal injuries through an ergonomic modification [34] and a multifaceted injury prevention program [35].

Research Gaps

As summarized above, research gaps in the field intersecting MSDs, healthy buildings, and the hotel industry appear to be numerous. Specifically, long-term outcomes following interventions aimed at mitigating MSDs are suboptimal, and innovative strategies should be explored [11]. Also, previous research on healthy building determinants and MSDs found minimal literature assessing interventions and the relationships between healthy building determinants and MSDs within the hotel industry. Moreover, the epidemiology of MSDs across a wide variety of hotel occupations, the influence of hotels' indoor built environment on employees' and guests' musculoskeletal health, and evidence-based solutions for MSD management within the hotel industry have not been comprehensively examined.

Objective

The objective of this paper is to provide a narrative review of the literature and a critical reflection on MSDs and healthy buildings in the hotel industry. Previous work [11,19,22] and the initial literature searches for this project did not uncover any peer-reviewed studies examining the aggregate interface of MSDs, healthy buildings, and hotels. Hence, this paper focuses on two areas: MSDs in hotel employees and MSD services for hotel guests. Based on the findings of the evidence synthesis, this paper proposes recommendations to address gaps in knowledge, research, policy, and practice regarding MSDs and healthy buildings in the hotel industry.

## Review

Materials and methods

Overview

A review of the literature was conducted using narrative overview and best-evidence synthesis processes [36]. Given the early stage of research for this field, a narrative review was undertaken with a broad objective and without a focused research question, which accommodated a wide range of eligible studies and heterogeneous outcome measures. With such an approach, a systematic review was not considered to be ideal. Hence, the analysis and evidence synthesis procedures used in this review were descriptive in nature, and quantitative analysis (e.g., meta-analysis) was not conducted. Nevertheless, for reproducibility of methods, systematic approaches for reporting were incorporated as appropriate, such as those recommended by the Preferred Reporting Items for Systematic Reviews and Meta-Analyses (PRISMA) [37].

Eligibility Criteria

Study eligibility criteria are defined using the PICOTS approach [11,37], as illustrated in Table 2.

**Table 2:** Study eligibility criteria

Domain	Description
P – Patient/population	Inclusion: Examined humans aged 18 years and older with MSDs, which are “diverse conditions affecting bones, joints, muscles, and connective tissues” [2]. MSDs could involve the spine, extremities, or general body regions (e.g., certain types of arthritis) [1,11]. Examined any type, intensity, and duration of MSDs. Exclusion: Examined systemic (e.g., rheumatoid arthritis, fibromyalgia) and neurological disorders (e.g., Parkinson’s disease, multiple sclerosis).
I – Intervention/exposure	Inclusion: Assessed eight exposures related to healthy building determinants: air quality and ventilation, dust and pests, lighting and views, moisture, noise, safety and security, thermal health, and water quality [11,18]. Assessed MSDs in hotel settings or interventions for the prevention and treatment of MSDs delivered in hotel settings.
C – Comparator	Inclusion (for interventional studies): Assessed interventions for the prevention and treatment of MSDs delivered in non-hotel settings (e.g., clinician offices) and compared them with hotel-based delivery.
O – Outcomes/variables	Inclusion: Used various approaches to measure healthy building determinants and MSDs, including patient-reported outcome measures, physical fitness and functional tests, and environmental outcomes. Measured outcomes directly pertinent to MSDs (e.g., pain, disability, absenteeism). Exclusion: Assessed indirect measures (e.g., body mass index, lifestyle elements, psychosocial factors) [11].
T – Time/timing	Inclusion: Cited in PubMed from database inception through November 15, 2024, or published in a Quartile 1-ranked journal in the “Tourism, Leisure and Hospitality Management” category of SCImago from journal inception through April 2, 2025.
S – Setting	Inclusion: Assessed MSDs or delivered MSD-related services within the indoor built environment of hotel settings [11]. A hotel is “an establishment that provides lodging and usually meals, entertainment, and various personal services for the public” [38].
Other eligibility criteria	Inclusion: Peer-reviewed; abstract available for screening and full-text article available in English; and included any subject-level original research design, except case reports. Exclusion: Only reported biomechanical modeling or qualitative data; lacked direct MSD measures; lacked human data; did not assess the independent effect of the hotel industry; or did not perform statistical comparisons.

Information Sources and Search Strategy

Studies were discovered by searching the peer-reviewed literature from medical and hospitality sources. For the medical literature, PubMed was searched using a strategy developed by the authors for MSDs, healthy buildings, and the hotel industry (Table 3). For the hospitality literature, the top 25% ranked (Quartile one) journals in the "Tourism, Leisure and Hospitality Management" category of the SCImago Journal & Country Rank of the Scopus database were searched for relevant articles [39]. Each journal was searched using the search feature on the journal's website using relevant terms, such as "healthy buildings," "healthy building," "musculoskeletal," and "back pain." Additionally, hand searches were conducted of citations within eligible studies and systematic reviews from the formal searches, as well as the authors' available files.

**Table 3:** PubMed search strategy

Search number	Search terms	Results
4	1 AND 2 AND 3	2,047
3	"hospitality"[All Fields] OR "hotel"[All Fields] OR "hotels"[All Fields] OR "hostel"[All Fields] OR "hostels"[All Fields] OR "motel"[All Fields] or "motels"[All Fields]	28,037
2	"healthy buildings"[All Fields] OR "healthy building"[All Fields] OR "sick building syndrome"[MeSH Terms] OR "sick building syndrome"[All Fields] OR "indoor environmental quality"[All Fields] OR "indoor environment"[All Fields] OR "environmental illness"[MeSH Terms] OR "environmental illness"[All Fields] OR "environmental illnesses"[All Fields] OR "built environment"[MeSH Terms] OR "built environment"[All Fields]	18,626
1	"back pain"[MeSH Terms] OR "back pain"[All Fields] OR "neck pain"[MeSH Terms] OR "neck pain"[All Fields] OR "radiculopathy"[MeSH Terms] OR "radiculopathy"[All Fields] OR "radiculopathies"[All Fields] OR "sciatica"[MeSH Terms] OR "sciatica"[All Fields] OR "sciaticas"[All Fields] OR ("musculoskeletal"[All Fields] AND "conditions"[All Fields]) OR "musculoskeletal conditions"[All Fields] OR ("musculoskeletal"[All Fields] AND "condition"[All Fields]) OR "musculoskeletal condition"[All Fields] OR ("musculoskeletal"[All Fields] AND "disorders"[All Fields]) OR "musculoskeletal disorders"[All Fields] OR ("musculoskeletal"[All Fields] AND "disorder"[All Fields]) OR "musculoskeletal disorder"[All Fields] OR "musculoskeletal pain"[MeSH Terms] OR ("musculoskeletal"[All Fields] AND "pain"[All Fields]) OR "musculoskeletal pain"[All Fields] OR ("musculoskeletal"[All Fields] AND "pains"[All Fields]) OR "musculoskeletal pains"[All Fields] OR "musculoskeletal diseases"[MeSH Terms] OR ("musculoskeletal"[All Fields] AND "diseases"[All Fields]) OR "musculoskeletal diseases"[All Fields] OR ("musculoskeletal"[All Fields] AND "disease"[All Fields]) OR "musculoskeletal disease"[All Fields] OR "musculoskeletal system"[MeSH Terms] OR ("musculoskeletal"[All Fields] AND "system"[All Fields]) OR "musculoskeletal system"[All Fields] OR ("musculoskeletal"[All Fields] AND "systems"[All Fields]) OR "musculoskeletal systems"[All Fields]	2,538,394

Selection Process

Articles obtained from the searches were handled in a similar manner to other work [11]. Briefly, results from the formal searches were exported to a citation manager and spreadsheets. The authors screened the articles for relevance and determined eligibility. No automation tools were used in the selection process.

Risk of Bias Assessment

Two 14-item instruments from the U.S. National Institutes of Health (NIH) were used to assess study quality (risk of bias); one was for cross-sectional and observational cohort studies, and the other for controlled interventional studies [40]. Items for the instruments are shown in the Appendix. Each item is scored as Yes (1) or No (0), and the sum of the 14 items results in a total score of 0-14. Overall study quality (risk of bias) ratings were derived from the total score as follows [11,41]: 0-4: Poor (high risk of bias); 5-9: Fair (between low and high risk of bias, some concerns about bias); 10-14: Good (low risk of bias).

Study evidence level was classified with methods adapted from the Oxford Centre for Evidence-Based Medicine [11,41-43]. Reporting bias related to missing results was not assessed because of the quantity and quality of evidence uncovered in the searches [11,41]. Meta-analysis was not conducted because of the aim of a narrative review and the heterogeneity of outcome measures used in the eligible studies. Likewise, publication bias was not examined through formal methods such as Egger's or funnel plot tests [44]. Nonetheless, inferences about publication bias were made by reviewing differences in the reported study outcomes (positive versus negative) according to risk of bias levels and sample sizes [41].

Evidence Synthesis

The analysis and evidence synthesis procedures used in this review were descriptive in nature. Given the early stage of this field, a narrative review was undertaken with a broad aim, which allowed for a wide range of eligible studies and heterogeneous outcome measures. Thus, descriptive synthesis was carried out, and quantitative analysis (e.g., meta-analysis) was not. For the descriptive synthesis, pertinent data were extracted from eligible articles and other information sources and summarized in evidence tables and narrative text. The authors synthesized the best available evidence and interpreted findings. Once a consensus regarding the evidence was reached, recommendations were made for medical and hotel stakeholders for research and implementation efforts, as well as healthcare value proposition development.

Results

Study Selection

**Figure 1:**
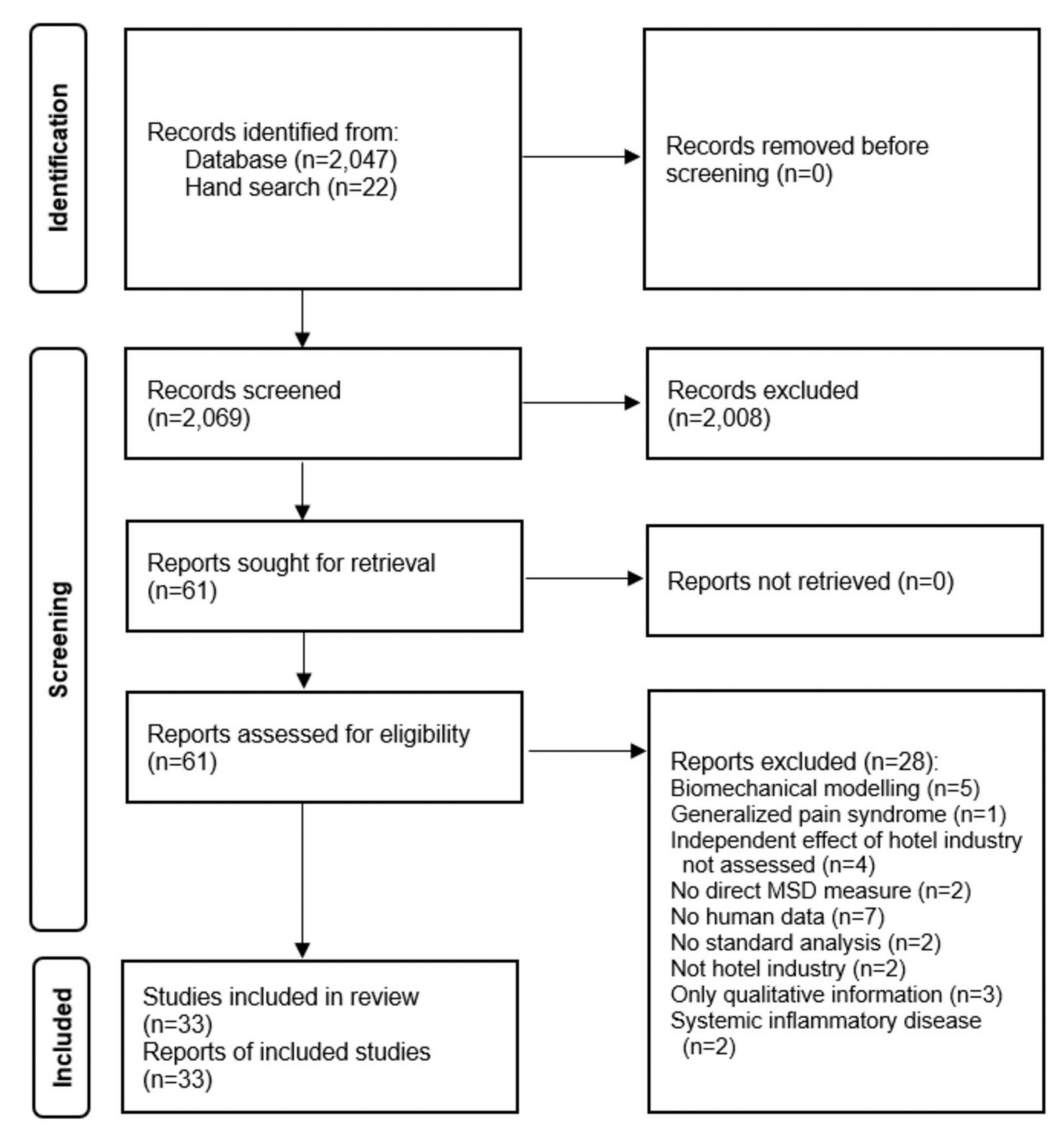
PRISMA diagram depicting PubMed search results PRISMA, Preferred Reporting Items for Systematic Reviews and Meta-Analyses

A PRISMA diagram of the PubMed search results is depicted in Figure 1. Thirty-three eligible studies were found [35,45-76]. Twenty-eight studies deemed relevant or of uncertain relevance at initial screening were found to be ineligible upon review of full-text articles.

Study Characteristics

A total of 64,998 participants were enrolled in the 33 eligible studies. Studies were conducted in 14 countries, with the most common locations being the United States (n=8) [35,46-48,57,60,67,69], India (n=5) [49,55,64,71,72], and France (n=4) [51,54,56,62]. Twenty-one studies were cross-sectional [45,47-50,55,57,60,61,64-72,74-76], while 12 were interventional [35,46,51-54,56,58,59,62,63,73], including six randomized controlled trials [51,53,54,56,58,62], three prospective cohorts [52,63,73], and three retrospective cohorts [35,46,59]. None of the cross-sectional studies evaluated MSDs in hotel guests or the three-way interaction among MSDs, healthy building determinants, and the hotel industry. None of the interventional studies evaluated the impact of modifying healthy building determinants on MSDs or the three-way interaction among MSDs, healthy building determinants, and the hotel industry.

For the cross-sectional studies (Table 4), relationships among MSDs and several types of hotel workers were assessed, which primarily examined MSD frequency (e.g., prevalence, incidence) in the target population. Some studies assessed physical function, disability, and other work-related factors. Fourteen studies assessed several types of MSDs [45,49,50,55,57,60,64-70,72], three assessed back pain [71,75,76], two assessed general MSDs [47,61], one assessed upper extremity MSDs [48], and one assessed neck pain and upper extremity MSDs [74]. Twelve studies assessed hotel housekeepers [48,57,60,64-70,74,75], three assessed hotel kitchen workers [45,71,72], three assessed general or various hotel workers [47,55,61], two assessed hotel restaurant workers [50,76], and one assessed hotel receptionists [49].

**Table 4:** Characteristics and outcomes of included cross-sectional studies ANCOVA, analysis of covariance; ANOVA, analysis of variance; BMI, body mass index; ERI, effort-reward imbalance; F, female; M, male; MSD, musculoskeletal disorder; MSK, musculoskeletal; NA, not applicable; NMQ, Nordic Musculoskeletal Questionnaire; NR, not reported; OA, osteoarthritis; ODI, Oswestry Disability Index; QOL, quality of life; ROM, range of motion; VAS, visual analog scale; WOMAC, Western Ontario and McMaster Universities Osteoarthritis Index; WRSP, work-related shoulder pain; yr, year

(Author, year)	Population studied, sample size (gender), age, country	Eligibility criteria	MSD region. Outcome measure. AQanalysis	Results
Abdelsalam, 2023 [45]	Hostel kitchen workers, 128 (43 F, 85 M), 44.8±8.2 yr, Egypt	Inclusion: Full-time kitchen workers from 2 hostels at an Egyptian university. Exclusion: NR.	MSD - various. Pain: NMQ. Work-related: various. Chi-square, T-test, regression.	90.6% of kitchen workers reported MSDs in the past 12 months, with most common regions being the low back (64.8%), knee (46.9%), foot (46.1%), neck (29.7%), shoulder (23.4%). Report of MSD was significantly related (p<0.05) to age, education level, job type and duration, BMI, but not related to various other work-related factors.
Buchanan, 2010 [47]	Hotel workers, 55,327 (24,048 F, 31,135 M), NR, USA	Inclusion: Unionized workers from 50 hotels, occupational injury data available. Exclusion: NR.	MSD - general. Work-related: MSK injury claims data. MSK injury rate, risk ratio, regression.	MSK injury rate in hotel workers was 5.2 injuries per 100 worker-years. Rate was highest in housekeepers (7.9), followed by dishwashers (6.0), cooks/kitchen workers (6.0), other jobs (4.9), servers (2.8). Injuries were significantly related (p<0.05) to age, gender (female), ethnicity (Hispanic), job type.
Burgel, 2010 [48]	Hotel housekeepers, 493 (483 F, 10 M), 41.2±9.7 yr, USA	Inclusion: Housekeepers from 5 casino hotels. Exclusion: NR.	Upper extremity. Pain: Las Vegas Questionnaire. Work-related: ERI, job strain. Regression.	56% of hotel housekeepers reported WRSP in past 4 weeks. WRSP was significantly related (p<0.05) to ERI, but not related to job strain.
Chauhan, 2020 [49]	Hotel receptionists, 50 (22 F, 28 M), 20-30 yr (median), India	Inclusion: Receptionists from 11 5-star and 3-star hotels. Exclusion: <1 y experience as hotel receptionist.	MSD - various. Pain: NMQ. Work-related: Posture. MSD rate, Phi test.	In past 12 months, hotel receptionists reported pain in low back (94%), neck (94%), upper back (88%), calf (84%), ankle/foot (64%), knee (60%), hip/thigh (40%). Standing work posture was significantly related (p<0.05) to calf and foot/ankle pain, but not to low back, neck, upper back, hip/thigh, and knee pain.
Chyuan, 2004 [50]	Hotel restaurant workers, 905 (NR), 33.3±11.3 yr, Taiwan (China)	Inclusion: Restaurant workers from 24 5-star hotels. Exclusion: NR.	MSD - various. Pain: NMQ. MSD rate.	83.8% of hotel restaurant workers reported work-related MSK pain in past month, with most common regions being shoulder (57.9%), neck (54.3%), low back (52.7%), finger/wrist (46.5%), ankle/foot (42.8%).
Gawde, 2018 [55]	Hotel workers, 1,183 (NR), NR, India	Inclusion: Workers from 6 luxury hotels or semi-luxury hotels. Exclusion: NR.	MSD - various. Pain: Questionnaire. QOL: General health questionnaire. MSD rate, regression.	44.5% of hotel workers reported MSK pain for at least 2 weeks in past 6 months, with most common regions being back (27.0%), leg (17.4%), joints (13.3%), neck (7.4%). MSK pain was significantly related (p<0.05) to heavy lifting, psychological well-being, job type, and duration, but not related to various other work-related factors.
Hsieh, 2016 [57]	Hotel housekeepers, 27 (27 F, 0 M), 22-52 yr (range), USA	Inclusion: Latina housekeepers from 75 4-star, 3-star, 2-star, and 1-star hotels. Exclusion: NR.	MSD - various. Pain: Interview. MSD rate.	Most common regions of MSK pain reported by hotel housekeepers were low back (48%), neck (48%), upper back (48%), elbow/upper arm (44%), knee (41%).
Krause, 2005 [60]	Hotel housekeepers, 941 (931 F, 10 M), 41.7±9.6 yr, USA	Inclusion: Unionized hotel housekeepers. Exclusion: NR.	MSD - various. Pain: Questionnaire. Work-related: Various. MSD rate, regression.	Most common regions of severe or very severe spinal MSK pain in past month reported by hotel housekeepers were low back (63%), upper back (59%), neck (47%). Spinal MSK pain was significantly related (p<0.05) to physical work load and ergonomic problems, but not related to number of rooms cleaned or beds made per day.
Lee, 2013 [61]	Hotel workers, 1,016 (396 F, 620 M), 55-59 yr (median), South Korea	Inclusion: Workers from 5 hotels. Exclusion: NR.	MSD - general. Pain: NMQ. Work-related: Various. MSD rate, chi-square, regression.	23.1% of hotel workers reported MSK pain per NMQ time frames. In females, MSK pain was significantly related (p<0.05) to age, job type and intensity, shift schedule, and sleep satisfaction, but not related to various other work-related factors. In males, MSK pain was significantly related (p<0.05) to job type and sleep satisfaction, but not related to various other work-related factors.
Parmar, 2017 [64]	Hotel housekeepers, 40 (NR), NR, India	Inclusion: Hotel housekeepers from 3 cities. Exclusion: NR.	MSD - various. Pain: Interview. MSD rate.	25% of hotel housekeepers reported work-related MSDs during unspecified time period. Of those with MSDs, regions were low back (60%), knee/calf (30%), calf only (10%).
Rahman, 2017 [65]	Hotel housekeepers, 65 (26 F, 39 M), 21-40 yr (median), Malaysia	Inclusion: Housekeepers from 10 hotels. Exclusion: NR.	MSD - various. Pain: NMQ. MSD rate.	Most common regions of MSK pain reported by hotel housekeepers in past 12 months were low back (60%), wrist/hand (41.5%), knee (36.9%), upper back (30.8%).
Raji, 2020 [66]	Hotel housekeepers, 40 (21 F, 19 M), 32.8±11.2 yr, Malaysia	Inclusion: Housekeepers from 13 budget hotels, Malaysian, employed ≥1 yr, age 18-60 yr. Exclusion: Pregnant, heart disease, musculoskeletal surgery.	MSD - various. Pain: NMQ. MSD rate.	100% of hotel housekeepers reported MSDs in past 12 months, with most common regions being low back (77.5%), heel (57.5%), shoulder (52.5%), hand/wrist (50%), upper back (47.5%), neck (37.5%).
Rosemberg, 2019 [67]	Hotel housekeepers, 49 (49 F, 0 M), 40±11 yr, USA	Inclusion: Female; hotel housekeeper, age ≥18 yr. Exclusion: Private housekeepers and hotel housemen, bleeding disorders, unwilling to provide blood sample.	MSD - various. Pain: Questionnaire. MSD rate.	38.1% and 14.3% of housekeepers reported chronic back pain and chronic neck pain, respectively.
Sánchez-Rodríguez, 2022 [68]	Hotel housekeepers, 1,043 (1,43 F, 0 M), 43.3±10 yr, Spain	Inclusion: Hotel housekeepers from 39 primary healthcare centers, public health services card for Balearic Islands, age ≥18 yr. Exclusion: unable to go to public health center, no telephone, language barrier.	MSD - various. Pain: NMQ. Work-related: Various. MSD rate, chi-square, regression.	51% of hotel housekeepers reported chronic MSK pain in unspecified time period, with most common regions being low back (28.7%), hand/wrist (23.7%), neck (21.6%), shoulder (19.9%). MSK pain was significantly related (p<0.05) to age, work duration, beds made per day, difficulty pushing cart, but not related to BMI and physical activity.
Shapoval, 2022 [69]	Hotel housekeepers, 140 (129 F, 11 M), 40-69 yr (median), USA	Inclusion: Unionized hotel housekeepers, immigrant. Exclusion: NR.	MSD - various. Pain: Questionnaire. MSD rate.	Of hotel housekeepers reporting work-related MSK injuries in unspecified time period, most common regions of severe to very severe MSK pain were low back (32.4%), upper back (31.9%), neck/shoulder (28.5%), knee (25.1%).
Silva, 2012 [70]	Hotel housekeepers, 18 (17 F, 1 M), 40 yr (median), Brazil	Inclusion: Housekeeper from national hotel chain, recurrent MSDs over a 1-yr period. Exclusion: NR	MSD - various. Pain: Questionnaire. MSD rate.	Of hotel housekeepers reporting MSK pain in past 12 months, most common regions were back (38.9%), upper limb (22.2%), neck (16.7%).
Shankar, 2015 [71]	Hostel kitchen workers, 114 (0 F, 114 M), 26.4±7.7 yr, India	Inclusion: Male, commercial kitchen worker from 9 hostel kitchens at 1 college. Exclusion: NR.	Low back. Pain: NMQ. Work-related: Various. MSD rate, chi-square.	65.8% of hostel kitchen workers reported low back pain in past 12 months. Low back pain was significantly related (p<0.05) to age, job type and duration, marital status, repetitive work, excessive force during cooking, but not related to various other work-related factors.
Subramaniam, 2015 [72]	Hostel kitchen workers, 114 (0 F, 114 M), 26.4±7.7 yr, India	Inclusion: Male, commercial kitchen worker from 9 hostel kitchens at a college. Exclusion: NR.	MSD - various. Pain: NMQ. Work-related: Various. MSD rate, chi-square.	67.5% of hostel kitchen workers reported MSDs in past 12 months, with most common regions being low back (65.8%), shoulder (62.3%), finger/wrist (43.9%), knee/foot (42.1%, neck (38.6%). MSDs were significantly related (p<0.05) to job type and duration, marital status, repetitive work, excessive force during cooking, but not related to various other work-related factors.
Wami, 2019 [74]	Hotel housekeepers, 422 (388 F, 34 M), 26.7±4.9 yr, Ethiopia	Inclusion: Male, hotel housekeeper, employed ≥1 yr. Exclusion: Spinal deformity, accident impacting musculoskeletal system.	Neck, upper extremity. Pain: NMQ. Work-related: Various. MSD rate, chi-square, regression.	62.8% of hotel housekeepers reported neck and upper limb MSDs in past 12 months, with most common regions being shoulder (54%), neck (50.7%), elbow/forearm (47.2%), hand/wrist (45.5%). Neck and upper limb MSDs were significantly related (p<0.05) to age, rest breaks, repetitive movement, reaching/overstretching, organization concern, and job satisfaction, but not related to various other work-related factors.
Wami, 2019 [75]	Hotel housekeepers, 422 (388 F, 34 M), 26.7±4.9 yr, Ethiopia	Inclusion: Male, hotel housekeeper, employed ≥1 yr. Exclusion: Spinal deformity, accident impacting musculoskeletal system.	Low back. Pain: NMQ. Work-related: Various. MSD rate, chi-square, regression.	58.1% of hotel housekeepers reported low back pain in past 12 months. Low back pain was significantly related (p<0.05) to employment status, rest breaks, reaching/overstretching, repetitive bending, job satisfaction, health training, beds made per day, but not related to various other work-related factors.
Yalew, 2022 [76]	Hotel restaurant waiters, 420 (257 F, 163 M), 17-53 yr (range), Ethiopia	Inclusion: Hotel restaurant waiter, employed for ≥1 yr. Exclusion: spinal deformity, traumatic or medically diagnosed low back pain, back surgery.	Low back. Pain: NMQ. Work-related: Various. MSD rate, regression.	43.8% of hotel restaurant waiters reported low back pain during their career. Low back pain was significantly related (p<0.05) to gender, regular exercise, prolonged standing at work, repetitive work tasks, but not related to marital status, or additional part of job.

For the interventional studies (Table 5), 10 studies assessed healthcare services delivered within hotel settings for the general population (i.e., hotel guests) with MSDs [51-54,56,58,59,62,63,73]. In these studies, some form of spa therapy with or without other interventions delivered in hotels was typically compared to a minimal intervention control delivered outside of hotels. Five of these studies assessed knee osteoarthritis [52-54,58,63], four assessed low back pain [51,56,62,73], and one assessed various types of osteoarthritis [59]. Additionally, two interventional (retrospective cohort) studies assessed MSDs in hotel employees [35,46]. Specifically, one study compared specialty care with discounted-fee care for the management of hotel workers with work-related MSDs [46]. Another study without a control group assessed a work-site MSD injury prevention program in hotel workers [35].

**Table 5:** Characteristics and outcomes of included interventional studies ANCOVA, analysis of covariance; ANOVA, analysis of variance; BMI, body mass index; F, female; M, male; MSD, musculoskeletal disorder; MSK, musculoskeletal; NA, not applicable; NMQ, Nordic Musculoskeletal Questionnaire; NR, not reported; OA, osteoarthritis; ODI, Oswestry Disability Index; QOL, quality of life; ROM, range of motion; VAS, visual analog scale; WOMAC, Western Ontario and McMaster Universities Osteoarthritis Index; yr, year

(Author, year), Population studied, Sample size (gender), age, country	Study type. Eligibility criteria	Intervention	Control	MSD region. Outcome measure. Analysis	Results
Atcheson, 2001 [46], Hotel-casino employees, 383 (198 F, 185 M), 40.8 yr, USA	Retrospective cohort. Inclusion: Employee of 2 large hotel-casinos, workers' compensation claim for MSD in 1995-1996. Exclusion: NR.	Specialist direct care: Coordinated by in-house musculoskeletal specialists. Referral to various outside specialists, as needed. Intervention details: NR.	Discounted fee system care: No in-house musculoskeletal specialists. Referral to single panel of providers selected by benefits administrator. Intervention details: NR.	MSD - various. Cost: Total, medical, indemnity costs. Prevalence/Incidence: Number (%) of participants with reported musculoskeletal injuries. ANOVA, chi-square.	At follow-up, mean costs per claim for total, medical, and indemnity were significantly lower (p<0.05) for intervention compared with control. No significant difference between groups for number (%) of reported musculoskeletal injuries.
Constant, 1995 [51], General population with low back pain, 126 (94 F, 32 M), 52 yr, France	Randomized controlled trial. Inclusion: Low back pain ≥1 yr, live within 40 km of spa resort. Exclusion: Contraindications to spa therapies, various internal disorders, lumbar surgery, sciatica.	1. Balneotherapy with underwater flow (36°C, 10 min). Local mud application (45°C, 20 min). High-pressure shower (36°C, 2.5 min). 1X/day, 6 days/week, 3 weeks. Delivered at hotel. 2. Control intervention: Pain medications, as needed. 3 weeks.	Pain medications, as needed. 3 weeks.	Low back. Pain: VAS. Disability: RMDQ. Function: Trunk flexion ROM. Medication usage: NSAIDs, analgesics. ANCOVA.	At 3-week and 6-month follow-up, significantly greater improvements (p<0.05) in pain, disability, function, and medication usage were observed in intervention compared with control.
Elkayam, 1991 [52], General population with knee osteoarthritis, 12 (11 F, 1 M), 51-70 yr (range), Israel	Prospective cohort (for group with osteoarthritis). Inclusion: Knee osteoarthritis symptomatic for ≥6 months, moderate-severe osteoarthritis per radiography. Exclusion: Uncontrolled hypertension, heart disease, peripheral vascular disease.	Balneotherapy (38°C, 20 min, 1X/day). Mud pack to knee (45°C, 20 min every other day). NSAIDs. 2 weeks. Delivered at hotel.	NA	Knee. Pain: Severity Rating Scale. Disability: Knee OA Severity Index. ANOVA	At 24-week follow-up, significant (p<0.05) reductions in knee OA severity, night pain, tenderness, and motion pain were observed compared with baseline.
Fioravanti, 2012 [53], General population with knee osteoarthritis, 60 (30 F, 30 M), 70.9±7.4 yr, Italy	Randomized controlled trial. Inclusion: Knee osteoarthritis symptomatic for ≥3 months, pain ≥30/100, Kellgren radiological score 1-3. Exclusion: Various internal disorders, cancer, pregnant, nursing baby.	1. Balneotherapy (38°C, 20 min, 1 session/day, 12 sessions, 2 weeks). Delivered at hotel. 2. Control intervention: Pain medications, as needed. Continued prescribed pharmacological and non-pharmacological interventions. 2 weeks.	Pain medications, as needed. Continued prescribed pharmacological and non-pharmacological interventions. 2 weeks.	Knee. Pain: VAS. Pain/Disability: Knee OA Severity Index. Disability/Function: WOMAC. QOL: SF-36. Medication usage: NSAIDs, analgesics. Proportion test, Mann-Whitney test, T-test.	At 12-week follow-up, significantly greater improvements (p<0.05) in pain, disability, function, QOL, and medication usage were observed in intervention compared with control.
Forestier, 2010 [54], General population with knee osteoarthritis, 451 (214 F, 237 M), 63.6±9.8 yr, France	Randomized controlled trial. Inclusion: Knee osteoarthritis symptomatic for ≥3 months, pain ≥30/100, radiographic evidence of knee osteoarthritis, age >50 yr or morning knee stiffness or knee crepitation. Exclusion: Various internal disorders, cancer, contraindications to spa therapy, previous specified medical treatment for knee osteoarthritis.	1. Balneotherapy (37°C, 15 min). Lower extremity massage under mineral water (38°C, 10 min). Mud pack to knee (37°C, 15 min). Supervised general aquatic therapy in mineral water (32°C, 25 min). 6 days/week, 3 weeks. Delivered at hotel. 2. Control intervention: Home exercise program of 4 exercises (6 sets, 3X/day). Prescribed pain medications and physical therapy. 3 weeks.	Home exercise program of 4 exercises (6 sets, 3X/day). Prescribed pain medications and physical therapy. 3 weeks.	Knee. Pain: VAS, Disability/Function: WOMAC. Chi-square, T-test.	At 6-month follow-up, significantly greater improvements (p<0.05) in pain, disability, and function, as well as % of patients achieving minimal clinically important improvements, were observed in intervention compared with control.
Guillemin, 1994 [56], General population with low back pain, 104 (63 F, 41 M), 58.3±2.3 yr, France	Randomized controlled trial. Inclusion: Low back pain ≥2 yr, erythrocyte sedimentation rate <30 mm/h. Exclusion: Sciatica, lumbar disc herniation, back surgery, systemic inflammatory disease, various internal disorders, spa therapy in past year.	Balneotherapy: Underwater high-pressure shower (36°C, 15 min). Series of 3 min showers with various pressures (31-36°C). 1 session/day, 6 days/week, 3 weeks. Delivered at hotel.	Pain medications as prescribed by general practitioner. 3 weeks.	Low back. Pain: VAS. Disability: Waddell. Function: Trunk ROM. Medication Usage: NSAIDs, analgesics. Chi-square, T-test.	At 9-month follow-up, significantly greater improvements (p<0.05) in pain, function, and medication usage were observed in intervention compared with control. No significant difference between groups in disability.
Karagülle, 2007 [58], General population with knee osteoarthritis, 20 (17 F, 3 M), 60±9.8 yr, Turkey	Randomized controlled trial. Inclusion: Knee osteoarthritis that is functionally and radiographically severe. Exclusion: Balneotherapy in past year, various internal disorders, cancer, changes in therapy in past 2 months, knee injections in past 6 months.	1. Balneotherapy (38°C, 30 min). 2x sessions/day, 10 days. 2. Therapeutic massage (optional based on patient preference). 3. Use of other hotel facilities, e.g., relaxation, outdoor activities at seashore (optional based on patient preference). 10 days. Delivered at hotel.	Pain medications as prescribed. 10 days.	Knee. Pain: VAS. Pain/Disability: Knee OA Severity Index. Function: Stair, walk, squat. Mann-Whitney U test.	At 24-week follow-up, significantly greater improvements (p<0.05) in pain and disability were observed in intervention compared with control. No significant difference between groups in function.
Karagülle, 2016 [59], General population with osteoarthritis, 239 (152 F, 87 M), 72.3±5.6 yr, Turkey	Retrospective cohort. Inclusion: Osteoarthritis, age ≥65 yr, prescribed spa therapy between March 7, 2002 and December 31, 2012. Exclusion: Other joint diseases, such as inflammatory arthritis.	Balneotherapy (38-40°C, 10-30 min, 1X/day) with or without: massage, exercise, Turkish bath. 2 weeks. Delivered at hotel.	NA	MSD - various. Pain: VAS. Disability: Health Assessment Questionnaire Disability Index. T-test.	At follow-up, significant reductions (p<0.05) in pain and disability were observed compared with baseline.
Landers, 2004 [35], Hotel housekeeping supervisors, housekeepers, guest room attendants, 450 (NR), NR, USA	Retrospective cohort. Inclusion: Hotel housekeeping supervisors, housekeepers, guest room attendants from 1 large hotel. Exclusion: NR.	Worksite education and participant practice on lifting, posture, exercises, other injury prevention topics (2 hours/session, 2 sessions). Instructions to supervisors to lead daily exercise and injury prevention activities for employees. Practical exam. Worksite ergonomics modification. Instructions for those deemed at injury risk or placed in light-duty work.	NA	MSD - general. Work-related: Total injury claims (# claims), lost work time (days), restricted duty (days). Cost: medical expenses ($). Percentage reduction compared to benchmark year.	At year 2, 62%, 97%, 56%, and 88% reductions in total injury claims, lost work time, restricted duty, and medical expenses, respectively, were observed compared with benchmark year.
Nguyen, 2017 [62], General population with low back pain, 87 (51 F, 36 M), 47.0 (37.0, 53.0) yr, France	Randomized controlled trial. Inclusion: Subacute-chronic low back pain or low back pain with radicular pain, age 18-60 yr, sick leave 4-24 wk. Exclusion: Cognition or behavioral disorder, contraindication to spa therapy.	1. Spa therapy consisting of balneotherapy (various parameters), aquatic exercise therapy, mud bath. 2 hours/session, 1 session/day, 5 days. 2. Educational program (4 sessions, 45 min/session, 4 sessions). 3. Educational booklet. Delivered at hotel. 5 days.	Usual medical care. Educational booklet. 5 days.	Low back. Pain: VAS. Disability: Quebec Back Pain Disability Scale. QOL: SF-12. Work-related: Return to work at 1 year, lost work time. Regression.	At follow-up, no significant differences were observed between groups in pain, disability, QOL, and work-related outcomes.
Odabaşı, 2002 [63], General population with knee osteoarthritis, 49 (35 F, 14 M), 60.5±6.2 yr, Turkey	Prospective cohort. Inclusion: Knee osteoarthritis according to Altman criteria. Exclusion: Hip or knee osteoarthritis, peripheral vascular disease.	Balneotherapy (39°C, 20 min, 1X/day), Mud bath (45°C, 20 min, 1X/day). Advice to not consume pain meds. 8 days. Delivered at hotel.	Balneotherapy (39°C, 20 min, 2X/day). Advice to not consume pain meds. 8 days. Delivered at hotel.	Knee. Pain: VAS. Pain/Disability: Knee OA Severity Index. Function: walk, squat, stairs. T-test.	At follow-up, significantly greater improvements (p<0.05) in pain, disability, and function were observed in intervention compared with control.
Tarcău, 2022 [73], General population with low back pain, 60 (35 F, 25 M), 53.1±11.1 yr, Romania	Prospective cohort. Inclusion: Low back pain for >3 months, lumbar disc protrusion. Exclusion: Indication for acute surgery, previous spine surgery at same level, spinal stenosis, radiculopathy, other spinal pathologies, chronic use of pain medications, various internal disorders.	1. Balneotherapy (37°C, 30 min). Core strengthening and flexibility exercises (45 min). 1X/day, 5 days/week, 2 weeks. Delivered at hotel. 2. Control intervention: Electrotherapy. 1X/day, 5 days/week, 2 weeks. Delivered at hotel.	Electrotherapy. 1X/day, 5 days/week, 2 weeks. Delivered at hospital.	Low back. Pain: VAS. Disability: ODI. Function: Trunk lateral flexion ROM, Trunk flexor and extensor strength. ANOVA.	At follow-up, significantly greater improvements (p<0.05) in pain and disability were observed in intervention compared with control. No difference between groups in function.

Risk of Bias

Study quality (risk of bias) for each study is found in Table 6 and Table 7. For the 21 cross-sectional studies (Table 6), the mean±SD score for study quality was 7.5±2.0 out of 14 (range 2-10, median eight), indicating a notable risk of bias. Two studies were of good quality (low risk of bias) [48,61], 17 were fair quality (some concerns about risk of bias) [45,47,49,50,55,60,65-72,74-76], and two were poor quality (high risk of bias) [57,64]. For the 12 interventional studies (Table 7), the mean±SD score for study quality was 9.0±2.1 out of 14 (range 6-11, median 8.5), indicating a notable risk of bias. This risk of bias was lower than in the cross-sectional studies, which may be due to the inherent shortcomings of cross-sectional designs. Five interventional studies were of good quality (low risk of bias) [46,53,54,58,62], and seven were fair quality (some concerns about risk of bias) [35,51,52,56,59,63,73]. Overall, many studies had weaknesses regarding a lack of details for sample size calculation, blinding, and handling of confounders. Similar deficits have been reported in other work [41]. No obvious publication bias was apparent when examining differences in study outcomes (positive versus negative) according to risk of bias levels and sample sizes.

**Table 6:** Study evidence level and quality (risk of bias) for cross-sectional studies Item number from the NIH study quality (risk of bias) assessment instrument [40]. Item scores of N, NA, and NR increase the risk of bias. Overall quality (risk of bias) rating [11,41]: 0-4: poor - high risk of bias (high). 5-9: fair - between low and high risk of bias (some: some concerns). 10-14: good - low risk of bias (low). N, no; NA, not applicable; NR, not reported; Y, yes; NIH, National Institutes of Health

		Study quality (risk of bias)																
		Item number																
Author, year	Evidence level	1	2	3	4	5	6	7	8	9	10	11	12	13	14	Total score	Study quality	Risk of bias
Abdelsalam, 2023 [45]	4. Cross-sectional	Y	Y	NR	Y	N	N	Y	Y	Y	N	Y	N	NA	Y	8	Fair	Some
Buchanan, 2010 [47]	4. Cross-sectional	Y	Y	Y	Y	N	N	Y	N	Y	N	Y	NR	NA	Y	8	Fair	Some
Burgel, 2010 [48]	4. Cross-sectional	Y	Y	Y	Y	N	N	Y	Y	Y	N	Y	Y	NA	Y	10	Good	Low
Chauhan, 2020 [49]	4. Cross-sectional	Y	Y	N	Y	N	N	Y	Y	Y	N	Y	NR	NA	Y	8	Fair	Some
Chyuan, 2004 [50]	4. Cross-sectional	Y	Y	Y	Y	N	N	Y	Y	Y	N	Y	Y	NA	N	9	Fair	Some
Gawde, 2018 [55]	4. Cross-sectional	Y	Y	N	Y	N	N	Y	Y	Y	N	Y	NR	NA	Y	8	Fair	Some
Hsieh, 2016 [57]	4. Cross-sectional	Y	Y	N	Y	N	N	Y	N	N	N	N	N	NA	N	4	Poor	High
Krause, 2005 [60]	4. Cross-sectional	Y	Y	Y	Y	N	N	Y	Y	Y	N	Y	NR	NA	Y	9	Fair	Some
Lee, 2013 [61]	4. Cross-sectional	Y	Y	Y	Y	N	N	Y	Y	Y	N	Y	Y	NA	Y	10	Good	Low
Parmar, 2017 [64]	4. Cross-sectional	Y	N	NR	N	N	N	Y	N	N	N	N	N	NA	N	2	Poor	High
Rahman, 2017 [65]	4. Cross-sectional	Y	Y	NR	Y	N	N	Y	Y	Y	N	Y	NR	NA	N	7	Fair	Some
Raji, 2020 [66]	4. Cross-sectional	Y	Y	Y	Y	N	N	Y	N	Y	N	Y	N	NA	N	7	Fair	Some
Rosemberg, 2019 [67]	4.Cross-sectional	Y	Y	NR	Y	N	N	Y	N	Y	N	Y	NR	NA	N	6	Fair	Some
Sánchez-Rodríguez, 2022 [68]	4. Cross-sectional	Y	Y	N	Y	N	N	Y	N	Y	N	Y	N	NA	Y	7	Fair	Some
Shapoval, 2022 [69]	4. Cross-sectional	Y	Y	N	Y	N	N	Y	Y	Y	N	Y	Y	NA	Y	9	Fair	Some
Silva, 2012 [70]	4. Cross-sectional	Y	Y	NR	Y	N	N	Y	N	N	N	Y	NR	NA	N	5	Fair	Some
Shankar, 2015 [71]	4. Cross-sectional	Y	Y	NR	Y	N	N	Y	N	Y	N	Y	NR	NA	Y	7	Fair	Some
Subramaniam, 2015 [72]	4. Cross-sectional	Y	Y	NR	Y	N	N	Y	N	Y	N	Y	NR	NA	Y	7	Fair	Some
Wami, 2019 [74]	4. Cross-sectional	Y	Y	Y	Y	Y	N	Y	N	Y	N	Y	N	NA	Y	9	Fair	Some
Wami, 2019 [75]	4. Cross-sectional	Y	Y	Y	Y	Y	N	Y	N	Y	N	Y	N	NA	Y	9	Fair	Some
Yalew, 2022 [76]	4. Cross-sectional	Y	Y	Y	Y	Y	N	Y	N	Y	N	Y	N	NA	Y	9	Fair	Some

**Table 7:** Study evidence level and quality (risk of bias) for interventional studies Item number from the NIH study quality (risk of bias) assessment instrument [40]. Item scores of N, NA, and NR increase the risk of bias. Overall quality (risk of bias) rating [11,41]: 0-4: poor: high risk of bias (high). 5-9: fair - between low and high risk of bias (some: some concerns). 10-14: good - low risk of bias (low). N, no; NA, not applicable; NR, not reported; Y, yes; NIH, National Institutes of Health; RCT, randomized controlled trial

			Study quality (risk of bias)																																	
			Item number																																	
Author, year	Evidence level		1		2		3		4		5		6		7		8		9		10		11		12		13		14		Total score		Study quality			Risk of bias
Atcheson, 2001 [46]		3. Retrospective cohort		Y		Y		Y		Y		N		N		Y		Y		Y		Y		Y		NR		Y		N		10		Good	Low	
Constant, 1995 [51]		2. RCT		Y		NR		NR		N		NR		Y		Y		Y		NR		Y		Y		N		Y		N		7		Fair	Some	
Elkayam, 1991 [52]		2. Prospective cohort		Y		Y		NR		Y		N		NR		Y		N		Y		N		Y		N		NR		N		6		Fair	Some	
Fioravanti, 2012 [53]		2. RCT		Y		Y		Y		N		Y		Y		Y		Y		NR		Y		Y		Y		Y		Y		12		Good	Low	
Forestier, 2010 [54]		2. RCT		Y		Y		Y		N		NR		Y		Y		Y		NR		Y		Y		Y		Y		Y		11		Good	Low	
Guillemin, 1994 [56]		2. RCT		Y		Y		NR		N		Y		N		Y		Y		NR		Y		Y		N		Y		N		8		Fair	Some	
Karagülle, 2007 [58]		2. RCT		Y		Y		Y		N		Y		Y		Y		Y		NR		Y		Y		N		Y		Y		11		Good	Low	
Karagülle, 2016 [59]		3. Retrospective cohort		Y		Y		Y		Y		N		N		Y		N		Y		N		Y		NR		NR		N		7		Fair	Some	
Landers, 2004 [35]		3. Retrospective cohort		Y		Y		NR		Y		N		N		Y		N		Y		Y		Y		NR		NR		N		7		Fair	Some	
Nguyen, 2017 [62]		2. RCT		Y		Y		Y		Y		Y		Y		N		Y		Y		Y		Y		N		Y		Y		12		Good	Low	
Odabaşı, 2002 [63]		2. Prospective cohort		Y		Y		NR		Y		Y		Y		Y		N		Y		N		Y		N		NR		N		8		Fair	Some	
Tarcău, 2022 [73]		2. Prospective cohort		Y		Y		NR		Y		Y		Y		Y		N		Y		N		Y		Y		NR		N		9		Fair	Some	

Summary of Available Evidence

Considering the substantial risk of bias found in the eligible studies, the findings discussed herein should be interpreted with caution. The available evidence indicates that hotel workers have high rates of and are at high risk for MSDs. One study comparing work-related musculoskeletal injury rates among various hotel workers reported that housekeepers have the highest rate (7.9 musculoskeletal injuries per 100 worker-hours), followed by dishwashers, cooks, and kitchen workers (6.0 musculoskeletal injuries per 100 worker-hours) [47]. Another study in hotel workers found that housekeepers have higher rates of chronic musculoskeletal pain than desk workers, but not other types of hotel workers [55]. Heavy lifting, high-intensity work, and high workload were found to be related to an increased risk of MSDs in hotel workers [55,60,61].

Low back pain appears to be the most common MSD in hotel workers. In housekeepers, frequencies range from 28.7% reporting chronic low back pain during an unspecified time period [68] to 77.5% reporting low back pain in the past 12 months [66]. In other hotel workers, frequencies range from 27% reporting low back pain for at least two weeks in the past six months [55] to 94% reporting low back pain in the past 12 months [49].

Six of the 12 interventional studies were controlled trials comparing hotel-based spa therapy to a control for the management of MSDs in the general population. For low back pain, one of the three studies administered guideline-based interventions (i.e., exercise, education) along with spa therapy [62], while the others did not [51,56]. For low back pain, the evidence was mixed. Namely, pain and functional outcomes for the spa therapy group were superior to the control in two of the three studies [51,56], while disability outcomes for the spa therapy group were superior to the control in one of the three studies [51]. For knee osteoarthritis, one of the three studies administered guideline-based interventions (i.e., exercise, massage) along with spa therapy [54]. The use of guideline-based interventions in the other two studies was unclear since they included such interventions as optional according to patient preference [53,58]. For knee osteoarthritis, the evidence generally supported spa therapy. Specifically, pain and disability outcomes for the spa therapy group were superior to the control in all three studies, while functional outcomes for the spa therapy group were superior to the control in two of the three studies [53,54].

Four of the remaining six interventional studies were observational cohorts that examined spa therapy with or without other interventions delivered in hotels for the management of MSDs in the general population [52,59,63,73]. The other two interventional studies examined the management of MSDs in hotel workers; one examined a work-site injury prevention program [35], and the other compared two types of care delivery models for the management of musculoskeletal injury workers' compensation claims [46]. Given the nature of these studies and the limited body of evidence, drawing conclusions from these data was not possible.

Hospitality Literature

Twenty-five articles were found in the hospitality literature. None of these articles examined the aggregate interface of MSDs, healthy buildings, and hotels, or otherwise met the review's inclusion criteria. However, they explored relevant topics that are noteworthy for the topic of this paper. One article modeled the impact of hotel building design features on the health and performance of hotel employees [77]. Two articles examined the built environment and human health in non-hospitality settings [78,79]. Six articles discussed various aspects of occupational health, wellness, and related domains in hotel employees and guests [80-85]; for example, the impact of back pain and other MSDs on presenteeism in hospitality employees [81], a summary of various injuries, such as MSDs, in hotel employees [82], an assessment of musculoskeletal injuries in hotel cooks [84], and a summary of MSDs and other occupational health conditions in hospitality workers [85].

Seven articles examined the use of hotels for healthcare services (e.g., "health tourism," "thermal tourism," "medical tourism") and related branding/marketing aspects [86-92], two of which mentioned back pain [87,88], and one discussed person-centered care [89]. Three articles discussed spa and resort therapies for health and wellness [93-95], which summarized the health benefits of spa therapies for MSDs [94], sleep quality in hotel guests [96], and consumption experience [97]. Three articles assessed the application of hospitality-type features in healthcare settings [98-100], while one article proposed a research agenda for occupational health in the hospitality industry [101].

Discussion

Key Findings - General Interpretation of Results

This paper is the first known attempt to collectively examine MSDs and healthy buildings through the lens of the hotel industry. It augments previous research that reported relationships among MSDs and healthy building determinants in the general population [11,19,22] by synthesizing the best available evidence in two areas with implications for the medical field and hotel industry: MSDs in hotel employees and MSD services in luxury resort hotels for hotel guests. Numerous gaps in knowledge, research, policy, and practice exist for the aggregate interface of MSDs, healthy buildings, and hotels, as illustrated below. Considering these gaps, along with the substantial risk of bias observed in the eligible studies, the available evidence should be interpreted with caution.

Many studies have assessed MSDs in hotel housekeepers, but limited evidence is available for other hotel workers. The available evidence suggests that hotel workers have high rates of MSDs, particularly low back pain, and the rate of MSDs appears to be highest for housekeepers. However, interventions to mitigate MSDs in hotel workers are lacking and are needed. Additionally, interventions for managing MSDs that are delivered in luxury resort hotels for guests may be beneficial for reducing pain and disability related to MSDs. However, research findings are mixed, and interventions for MSDs delivered in hotels do not appear to be fully aligned with evidence-based practices.

Limitations

The evidence uncovered in this review has knowledge gaps that preclude generalizability of findings and recommendations for solutions. For example, no peer-reviewed studies were found that assessed the combined impact of MSDs and healthy buildings in the context of the hotel industry, which is perhaps the biggest limitation of the available evidence. This research area is relatively new, and most of the available literature covers healthy buildings, MSDs, and hotels as separate domains. For the cross-sectional studies, only a few types of hotel workers were assessed, with housekeepers being the single type with sufficient data to make conclusions. Also, the impact of different types of hotel settings was not fully characterized, and the time periods used to describe MSD frequency varied widely, which made comparisons across studies difficult. Furthermore, the identified risk factors for MSDs in hotel workers were inconclusive, and no evidence was available to suggest that risk factors differ from similar occupations outside of the hotel industry. In addition, none of the studies reported on MSDs in hotel guests. Finally, costs and other economic variables were only examined in a few studies.

Less than half of the controlled trials assessing hotel-based therapies for MSDs administered guideline-based interventions. Additionally, the comparator (control) interventions in these trials were relatively weak and primarily consisted of minimal approaches or passive therapies. Considering these factors, it is possible that the positive findings observed in some trials for hotel-based spa therapies compared with control may be due to the environment or setting in which the care was delivered (luxury resort hotel versus medical) instead of specific interventional components. Furthermore, the interventions were delivered in luxury resort hotels, which limits generalizability to other types of hotels. Luxury resort hotels have features that enhance guest and patient experiences, which can facilitate musculoskeletal health programs. Nevertheless, it is plausible that accommodation fees in luxury resort hotels are cost-prohibitive for many people, and MSD services delivered in this setting are not covered by third-party payers.

Implications for Medical and Hotel Stakeholders

This paper has implications for medical and hotel stakeholders. Specifically, its findings can serve as starting points for the development of a new field by creating awareness, clarifying concepts, and providing preliminary recommendations about MSDs and healthy buildings in the hotel industry. However, considering that the evidence uncovered in this review has numerous gaps, it is not feasible to make specific recommendations about implementation strategies to directly change practice. As previously mentioned, this review found no peer-reviewed literature about the aggregate interface of MSDs, healthy buildings, and hotels, and previous research found minimal information about interventions aimed at healthy building determinants for MSDs. Given the substantial knowledge gaps, our recommendations rely, in part, on sources outside of the peer-reviewed literature, publicly available information, theoretical concepts, and experiences with relevant topics [23]. Furthermore, as the field grows from its infancy stage, it is important to examine how concepts, research evidence, and implementation factors from other occupational settings about MSDs and healthy buildings, separately or together, can be translated to the hotel industry.

Given the different perspectives of the various stakeholders, along with the infancy stage of this field, acting with caution is critical to avoid undesired outcomes, maximize the possibility of health benefits for hotel employees and guests, and augment financial return for the hotel industry. Additionally, medical and hotel stakeholders should work together to avoid the "silo" approach, foster collaboration, and optimize the advancement of this field. A similar approach has been suggested for medical and hospitality stakeholders for interdisciplinary research, [92] but has not been applied in the context of MSDs and the hotel industry.

If positive findings are observed in future research and implementation efforts, the desired long-term outcome of initiatives addressing MSDs and healthy buildings within the hotel industry is to deliver the right service to the right client, at the right time, at the right place, at the right price, and through the right channels [23]. In turn, this novel delivery approach should improve clinical outcomes and the health, wellness, quality of life, and experiential takeaway of people who utilize or work in hotels, as well as create a new healthcare value proposition for medical and hotel stakeholders.

Recommendations

Research: Clinical trials are needed to assess the following: effectiveness of evidence-based interventions delivered in luxury resort hotels on MSDs in hotel employees and guests; delivery of MSD services in hotels compared with clinician offices; and impact of addressing healthy building determinants on MSDs in hotel employees and guests. Furthermore, qualitative research with the general population and healthcare professionals is needed to examine preferences, attitudes, and expectations about receiving and delivering care for MSDs in luxury resort hotels versus clinician offices.

Large-scale epidemiological studies are needed to examine the prevalence of and risk factors for MSDs in hotel workers across various settings. Health economic evaluations are needed to study the impact of MSD and healthy buildings programs in the hotel industry across the hotel workforce, revenue from hotel guests, and delivery of MSD healthcare services in hotels. While the economic value of implementing healthy building designs has been evaluated in commercial real estate [102,103], it has not been assessed for healthy buildings in the hotel industry, particularly related to MSDs. Additionally, research efforts to develop, test, and implement healthy buildings certification systems for MSDs with applications for the hotel industry would be useful. Finally, to critically appraise the literature in the research areas discussed above, full-scale systematic reviews with meta-analyses would be beneficial.

Findings from the aforementioned research efforts should ideally be reported in both the hospitality literature and the medical literature. Medical stakeholders should review the literature published in hospitality journals and vice versa. This approach will encourage a comprehensive understanding and interprofessional collaboration for advancements in the field. However, the lack of interprofessional cooperation between medical and hospitality stakeholders has been previously reported [92]. Thus, these advancements will likely be a culture shift for which awareness and access barriers should be addressed. Similarly, expert panel meetings consisting of medical and hotel stakeholders should be conducted to formalize strategies and develop consensus statements for research initiatives.

Implementation: If positive findings are observed in future research efforts, the following principles are recommended for medical and hotel stakeholders to help implement products and services, advance the field in a direction aimed at increasing value-based care, mitigate risks, and avoid misconceptions and unnecessary controversies. Caution is advised regarding marketing claims, programs, and third-party commentaries that do not align with these principles. First, this new field should be built in an environment that fosters transparency, research integrity, public safety, ethical and moral behavior, and disclosure of potential conflicts of interest. Likewise, implementation efforts should focus on evidence-based practices that are driven by robust and pragmatic research initiatives. Moreover, adherence to regulatory standards is critical, such as those from healthcare professional licensing boards, the U.S. Federal Trade Commission, the U.S. Food and Drug Administration, and the U.S. Occupational Safety and Health Administration. The indoor built environment, facilities, products, and services should be structured according to recommendations of healthy buildings certifications for MSDs [23].

For this new field, avoiding undesired outcomes by carefully considering the potential harms associated with implementing healthcare products and services is crucial. For example, the hotel industry has recently embraced wellness concepts [31], and healthy building initiatives encourage active designs to foster physical activity, promote health, and reduce chronic disease [18,21,23,104,105]. Yet, individuals with MSDs may not use active design facilities because adherence to therapeutic exercise is often suboptimal [23,106-108]. Moreover, the specific components of exercise programs are important for MSD management [23], and safety must be considered, especially if exercise is performed without supervision [108]. Incorrect movements during exercise and other physical activities may aggravate pain and disability and delay recovery from MSDs [23,109]. Therefore, to avoid undesired outcomes for hotel employees and guests, safety and appropriate exercise prescription principles must be at the forefront of programs associated with MSD management and healthy buildings, such as active design.

Healthcare value proposition: An evidence-based healthcare value proposition "...reflects a comprehensive understanding of unmet needs, aligning with both operational efficiencies and economic value. It adapts to healthcare advancements and market demands, addressing the requirements of varied healthcare systems while responding to multifaceted challenges. It highlights the innovation’s value in improving clinical outcomes, system efficiency, and stakeholder benefits in a complex ecosystem" [110]. If positive findings are observed in future research and implementation efforts, the following recommendations should be considered when developing a healthcare value proposition for products and services related to MSDs and healthy buildings within the hotel industry.

First, the focus should be on clinical practice guidelines and evidence-based management for MSDs while embracing the experiential takeaway that guests receive within the hotel environment [111-115]. These aspects should be directed by an interprofessional team of healthcare practitioners who have appropriate clinical expertise, along with organizational support from hotel stakeholders who are reflective of the brand voice. Also, a thorough needs assessment and market analysis should be conducted for healthcare delivery systems associated with MSDs and healthy buildings within the hotel industry. Do these products and services clearly represent an innovation over existing approaches? Will these products and services improve clinical outcomes and the health, wellness, quality of life, and experiential takeaway of people who utilize or work in hotels? Are hotel guests willing to receive these services in hotels, and are healthcare practitioners willing to deliver them? Will the stakeholders in this market see these products and services as valuable, and are they willing to pay for them? Is a meaningful return on investment associated with the business case for these products and services? Assuming the answer is "Yes" to these questions, it is conceivable that healthcare delivery systems for MSDs and healthy buildings within the hotel industry could create a new healthcare value proposition.

An appropriate environment in hotel settings should be established to elicit the right emotions to foster adherence and accountability related to healthcare services for MSDs. Likewise, it would be useful to examine if aspects of service delivery in the hospitality industry, such as client-centered experiential service marketing and related concepts [111-115], could be applied to healthcare [23]. As a result, changing the customer's experience from the mental state of "I need to go there" (e.g., healthcare patients) to a more positive feeling of "I want to go there" (e.g., guests of luxury resort hotels) may help enhance clinical outcomes.

## Conclusions

In summary, this paper is the first known attempt to collectively assess MSDs and healthy buildings through the lens of the hotel industry. It augments previous research that reported relationships among MSDs and healthy building determinants in the general population. Findings indicate that there are numerous gaps in knowledge for the aggregate interface of MSDs, healthy buildings, and hotels; these gaps and the substantial risk of bias in the available evidence suggest that findings should be taken with caution; hotel workers are particularly vulnerable to MSDs, and interventions are needed to address MSDs in this occupational setting; and interventions delivered in luxury resort hotels may be useful for guests with MSDs.

Based on the available evidence and theoretical concepts, the recommendations proposed in this paper should help guide research and implementation efforts, as well as healthcare value proposition development, for medical and hotel stakeholders involved with this new field. Critically, initial endeavors should proceed with caution by assessing and addressing potential risks at each developmental phase. The proposed expansion of the MSD and healthy buildings field into the hotel industry is potentially an impactful value-based growth opportunity for patient care. If positive findings are observed in future research and implementation efforts, this novel delivery approach could improve clinical outcomes and the health, wellness, quality of life, and experiential takeaway of hotel employees and guests.

## Appendices

**Table 8:** NIH study quality (risk of bias) instrument for cross-sectional and observational cohort studies [34]

Item number	Description
1	Was the research question or objective in this paper clearly stated?
2	Was the study population clearly specified and defined?
3	Was the participation rate of eligible persons at least 50%?
4	Were all the subjects selected or recruited from the same or similar populations (including the same time period)? Were the inclusion and exclusion criteria for being in the study prespecified and applied uniformly to all participants?
5	Was a sample size justification, power description, or variance and effect estimates provided?
6	For the analyses in this paper, were the exposure(s) of interest measured prior to the outcome(s) being measured?
7	Was the timeframe sufficient so that one could reasonably expect to see an association between exposure and outcome if it existed?
8	For exposures that can vary in amount or level, did the study examine different levels of the exposure as related to the outcome (e.g., categories of exposure, or exposure measured as a continuous variable)?
9	Were the exposure measures (independent variables) clearly defined, valid, reliable, and implemented consistently across all study participants?
10	Was the exposure(s) assessed more than once over time?
11	Were the outcome measures (dependent variables) clearly defined, valid, reliable, and implemented consistently across all study participants?
12	Were the outcome assessors blinded to the exposure status of participants?
13	Was loss to follow-up after baseline 20% or less?
14	Were key potential confounding variables measured and adjusted statistically for their impact on the relationship between exposure(s) and outcome(s)?

**Table 9:** NIH study quality (risk of bias) instrument for controlled interventional studies [34]

Item Number	Description
1	Was the study described as a randomized, a randomized trial, a randomized clinical trial, or an RCT?
2	Was the method of randomization adequate (i.e., use of randomly generated assignment)?
3	Was the treatment allocation concealed (so that assignments could not be predicted)?
4	Were study participants and providers blinded to treatment group assignment?
5	Were the people assessing the outcomes blinded to the participants' group assignments?
6	Were the groups similar at baseline on important characteristics that could affect outcomes (e.g., demographics, risk factors, co-morbid conditions)?
7	Was the overall drop-out rate from the study at endpoint 20% or lower of the number allocated to treatment?
8	Was the differential drop-out rate (between treatment groups) at endpoint 15 percentage points or lower?
9	Was there high adherence to the intervention protocols for each treatment group?
10	Were other interventions avoided or similar in the groups (e.g., similar background treatments)?
11	Were outcomes assessed using valid and reliable measures, implemented consistently across all study participants?
12	Did the authors report that the sample size was sufficiently large to be able to detect a difference in the main outcome between groups with at least 80% power?
13	Were outcomes reported or subgroups analyzed prespecified (i.e., identified before analyses were conducted)?
14	Were all randomized participants analyzed in the group to which they were originally assigned, i.e., did they use an intention-to-treat analysis?
